# Development and Early Feasibility of Chatbots for Educating Patients With Lung Cancer and Their Caregivers in Japan: Mixed Methods Study

**DOI:** 10.2196/26911

**Published:** 2021-03-10

**Authors:** Yuki Kataoka, Tomoyasu Takemura, Munehiko Sasajima, Naoki Katoh

**Affiliations:** 1 Department of Respiratory Medicine Hyogo Prefectural Amagasaki General Medical Center Amagasaki Japan; 2 Department of Internal Medicine Kyoto Min-Iren Chuo Hospital Kyoto Japan; 3 School of Social Information Science University of Hyogo Kobe Japan

**Keywords:** cancer, caregivers, chatbot, lung cancer, mixed methods approach, online health, patients, symptom management education, web-based platform

## Abstract

**Background:**

Chatbots are artificial intelligence–driven programs that interact with people. The applications of this technology include the collection and delivery of information, generation of and responding to inquiries, collection of end user feedback, and the delivery of personalized health and medical information to patients through cellphone- and web-based platforms. However, no chatbots have been developed for patients with lung cancer and their caregivers.

**Objective:**

This study aimed to develop and evaluate the early feasibility of a chatbot designed to improve the knowledge of symptom management among patients with lung cancer in Japan and their caregivers.

**Methods:**

We conducted a sequential mixed methods study that included a web-based anonymized questionnaire survey administered to physicians and paramedics from June to July 2019 (phase 1). Two physicians conducted a content analysis of the questionnaire to curate frequently asked questions (FAQs; phase 2). Based on these FAQs, we developed and integrated a chatbot into a social network service (phase 3). The physicians and paramedics involved in phase I then tested this chatbot (α test; phase 4). Thereafter, patients with lung cancer and their caregivers tested this chatbot (β test; phase 5).

**Results:**

We obtained 246 questions from 15 health care providers in phase 1. We curated 91 FAQs and their corresponding responses in phase 2. In total, 11 patients and 1 caregiver participated in the β test in phase 5. The participants were asked 60 questions, 8 (13%) of which did not match the appropriate categories. After the β test, 7 (64%) participants responded to the postexperimental questionnaire. The mean satisfaction score was 2.7 (SD 0.5) points out of 5.

**Conclusions:**

Medical staff providing care to patients with lung cancer can use the categories specified in this chatbot to educate patients on how they can manage their symptoms. Further studies are required to improve chatbots in terms of interaction with patients.

## Introduction

Distress among patients with cancer and their caregivers has received increasing attention from medical staff. Early palliative care (EPC) from the time of diagnosis has been shown to have a positive impact on quality of life [1]. Through EPC, patients and their caregivers gain experience in several areas, including prompt and personalized symptom management [2].

Nonetheless, patient education remains a problem for health care providers [3]. Advancements in health care increase the amount of information that needs to be provided during patient education. Consequently, burnout among health care providers involved in EPC has become a problem [4].

Chatbots are artificial intelligence (AI)–driven programs that interact with people [5] through text messages and outputs on cellphone- or web-based platforms [6]. A simple rendition of this system involves a user entering text and AI predicting the corresponding predefined category and then sending the response corresponding to that category to the user. Considering the potential of this system to substitute the conversational mode of education that humans use and to save on labor, this technology has attracted increasing attention. Several chatbots are currently available for patients with breast cancer [7,8]; however, no chatbots are available for those with lung cancer and their caregivers. In this study, we aimed to develop and evaluate the early feasibility of a chatbot designed to answer questions of patients with lung cancer and their caregivers on a web-based platform whenever they experience unfamiliar symptoms; this would help them improve their knowledge of symptom management.

## Methods

### Study Design

To develop a chatbot for patients with lung cancer, we used a sequential mixed methods approach (Table 1) [9]. We adopted this approach because of the lack of sufficient nationwide data on the categories of frequently asked questions (FAQs) asked by patients with lung cancer and their caregivers. We also decided to adopt an initial qualitative approach to generate hypotheses. We adopted the lens of health care providers to provide palliative care. This study was conducted at a tertiary care hospital in Japan.

**Table 1:** Study overview.

Phase #	Procedures	Product	Evaluation method
1	Conducted a questionnaire-based survey with physicians and paramedics	Categories of FAQs^a^	Qualitative
2	Formulated responses	FAQ-response pairs	N/A^b^
3	Developed the chatbot	Chatbot version 1	N/A
4	Conducted an α test with the physicians and paramedics involved in phase 1	Chatbot version 2	Quantitative
5	Conducted a β test with patients with lung cancer and their caregivers	Chatbot version 3	Quantitative

^a^FAQs: frequently asked questions.

^b^N/A: not applicable.

#### Phase 1: Qualitative Survey

We conducted a web-based anonymized qualitative survey from June to July 2019. We included physicians in the Department of Respiratory Medicine at our institution, along with paramedics including nurses, pharmacists, physical therapists, occupational therapists, and clerks working with patients with lung cancer in the clinic, emergency department, or ward. We asked these health care workers to respond to the FAQs obtained from patients with lung cancer or their caregivers in an open-ended manner. We also referred to a previous study [10] and website [11] that categorized data obtained from telephone-based consultations with patients with lung cancer in Japan or their caregivers.

Two board-certified respiratory physicians (TT and YK) conducted a content analysis on the questions from the perspective of improving self-management of symptoms. TT generated these categories, and YK confirmed them. Disagreements were resolved through discussion between TT and YK.

#### Phase 2: Generation of Responses

Based on the categories generated in phase 1, TT generated the appropriate responses, and YK proofread them. Wherever necessary, staff members from the relevant departments were asked to review the responses. We then edited the responses on the basis of their comments.

#### Phase 3: Development of the Chatbot

**Figure 1:**
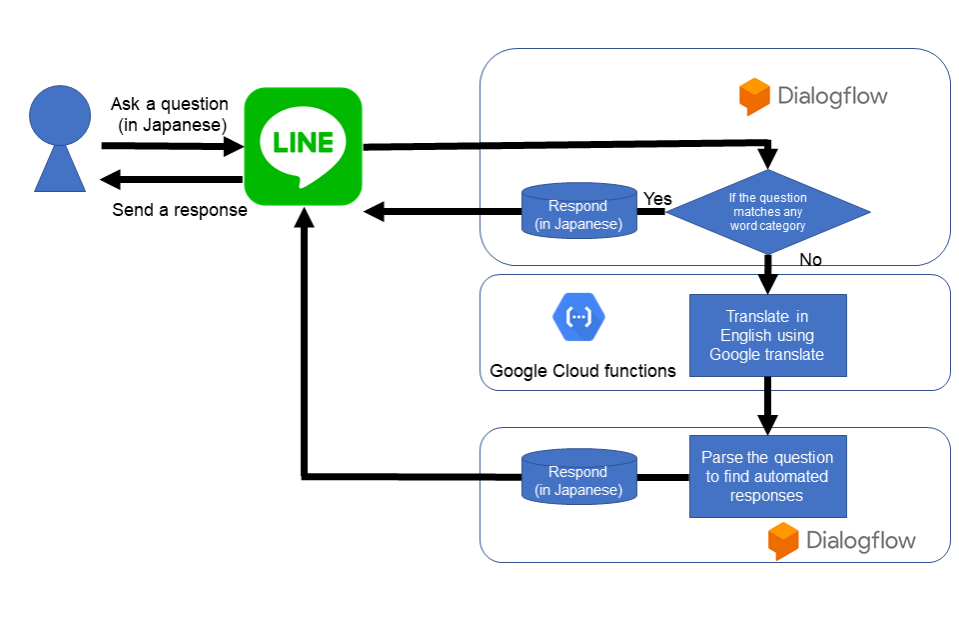
Chatbot architecture.

We decided to use Bot Designer (LINE Corp)—a bot designing service—owing to its ease of access, and the LINE social media platform to implement the chatbot. In Japan, LINE is the most popular social network service, being used by approximately 70% of the general population [12]. To generate automated responses, we used Google Cloud’s Dialogflow [13]. The architecture of this design is illustrated in Figure 1.

We adopted two natural language processing systems to match the responses. The following workflow process was followed: after receiving a question in Japanese from a user, Dialogflow determined whether the keywords included in the question matched the predetermined keywords for each category. If a match was identified, an appropriate response developed in phase 2 was sent to the patients via LINE. Otherwise, the text was translated into English and parsed to identify an appropriate category; thereafter, a response was sent to the patients.

#### Phase 4: α Test (Quantitative)

From May to June 2020, we invited the participants in phase 1 to an α test that involved the addition of keywords corresponding to the categories specified on the basis of the errors.

#### Phase 5: β Test (Quantitative)

From July to December 2020, we invited patients with lung cancer and their caregivers to the β test through paper flyers. Individual consent was obtained before registering the chatbot. We asked the participants to indicate their satisfaction levels with a 5-point Likert scale after the experiments (0=highly dissatisfied, 5=highly satisfied).

### Statistical Analysis

Descriptive statistics were used to summarize the results. We performed an unpaired *t* test for univariate analysis. We used Stata (version 16.1, Stata Corp) for statistical analysis.

### Ethical Consideration

The study protocol was approved by the institutional review board of the Hyogo Prefectural Amagasaki General Medical Center (number 1-31). We obtained individual consent from the participants through a web form.

## Results

### Phase 1: Qualitative Survey

We obtained 246 questions from 15 health care providers. We identified 5 major categories (home care: n=50, 20%, changes in health condition: n=54, 22%, prognosis: n=20, 8%, lifestyle: n=82, 33%, and medications: n=39, 16%), and 75 supplementary categories (Multimedia Appendix 1). We identified 7 additional categories by screening resources (Multimedia Appendix 2).

### Phase 2: Formulation of Responses

We formulated responses for 82 categories identified in phase 1. The responses contained brief information intended to teach patients and their caregivers to deal with symptoms and direct them to various weblinks to information resources that are maintained by the public budget.

### Phase 3: Development of a Chatbot

**Figure 2:**
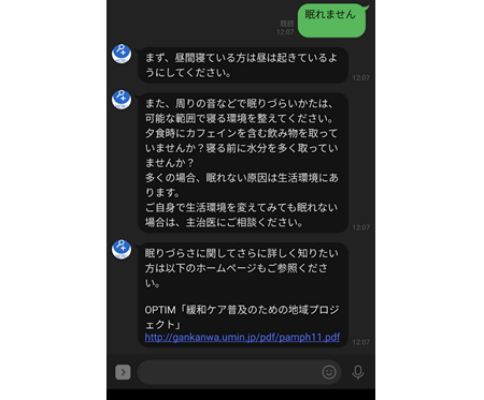
A sample conversation with the chatbot.

Based on the FAQs identified in phase 2, we developed the first version of the chatbot and integrated it in the LINE social media network. Figure 2 shows a sample interaction shared with the chatbot.

### Phase 4: α Test (Quantitative)

A total of 14 medical staff participated in the α test. They asked 71 questions to the chatbot during this period. Of these, 11 (15%) questions did not match the appropriate categories. Therefore, we added 3 new categories. Some of the categories had corresponding responses because the questions included the names of products associated with drinking and smoking. Thus, we added keywords for the corresponding categories to match responses.

### Phase 5: β Test (Quantitative)

From among 309 patients who received either oral or intravenous chemotherapy at our hospital, 11 patients and 1 caregiver participated in the β test. Of them, 9 patients were aged <60 years, 1 was aged between 60 and 70 years, and 2 were aged >70 years. All of them used computers or smartphones daily. The participants were asked 60 questions, of which 8 (13%) did not match with the appropriate categories. Furthermore, 2 (3%) questions had corresponding responses, and 6 (10%) questions did not. Additionally, 3 (5%) questions based on daily conversation did not have corresponding responses (eg, “I played golf today in the morning”).

After the test, 7 participants responded to the postexperimental questionnaire. The mean satisfaction score was 2.7 (SD 0.5) points out of 5. We used two free feedback comments: “those who have a connection with a patient group, the answers were not too much to hope for” and “there were many times when I did not get a proper answer.” We added 3 categories based on the unmatched questions. Finally, 91 categories and responses were curated [14].

## Discussion

### Principal Findings

This is the first study to develop a chatbot and evaluate its early feasibility to improve the knowledge of symptom management among patients with lung cancer and their caregivers. Based on the experience of medical professionals, we developed an autoresponsive chatbot, which could respond to most of the FAQs. However, it was not adequately used, and user satisfaction was low.

### Comparison with Previous Studies

The categories identified in this study could be used as a reference for patient education in future. Currently, only one chatbot is available to empower patients with cancer and their caregivers. This chatbot was developed only for patients with breast cancer [6,15,16]. Given that patients with breast cancer and those with lung cancer differ in their age of onset, chemotherapy regimens, and cancer-related symptoms [17], it is important to increase the number of categories to improve their knowledge of symptom management based on the FAQs identified in this study.

However, since the generation of the chatbot alone did not yield adequate questions from participants, the low response rate potentially indicates that our chatbot may not be acceptable in its current form. Further studies are required to activate the interaction between patients and chatbots. A previous systematic review reported that web-based interactions between physicians and patients or those among patients may improve the patients’ quality of life [18]. Thus, chatbots can be incorporated into web-based networks for increased interaction. Indeed, one of our patients involved in the β test (phase 5) preferred interactions with other patients rather than the chatbot. Therefore, further studies are required to generate a platform that initially forms a text messaging–based online support group involving medical professionals and patients and then gradually reduces the need for these professionals to respond to patient queries by involving chatbots instead.

### Limitations

This study has two major limitations of note. First, 8 (13%) questions did not match suitably with responses in phase 5, and a patient complained of not receiving appropriate responses. Although the ability of the chatbot to match questions with categories was not a major problem, questions for nonexistent categories remained unmatched. Therefore, it is necessary to add educational categories and responses through further discussion. Furthermore, the integration of existing chatbots for daily conversations may improve the proportion of matches [19]. Second, this was a single-center study, and further studies are needed to evaluate the applicability of our findings in other hospitals.

### Conclusions

Medical staff who provide care to patients with lung cancer may be able to educate them using a chatbot containing the categories of FAQs curated in this study. Nonetheless, further studies are required to improve this chatbot in terms of interaction, especially considering its low usage in this study.

## Appendix

Multimedia Appendix 1 Categories from the questionnaire.

Multimedia Appendix 2 Categories from the references.

## Abbreviations

AI: artificial intelligence
EPC: early palliative care
FAQ: frequently asked question
